# Non-invasive Transcranial Magnetic Stimulation (TMS) of the Motor Cortex for Neuropathic Pain—At the Tipping Point?

**DOI:** 10.5041/RMMJ.10130

**Published:** 2013-10-29

**Authors:** Roi Treister, Magdalena Lang, Max M. Klein, Anne Louise Oaklander

**Affiliations:** 1 Department of Neurology, Massachusetts General Hospital, Harvard Medical School, Boston, Massachusetts, USA;; 2 Department of Pathology (Neuropathology), Massachusetts General Hospital, Boston, Massachusetts, USA

**Keywords:** Chronic pain, clinical trial, motor cortex, neuropathic pain, transcranial magnetic stimulation (TMS)

## Abstract

The term “neuropathic pain” (NP) refers to chronic pain caused by illnesses or injuries that damage peripheral or central pain-sensing neural pathways to cause them to fire inappropriately and signal pain without cause. Neuropathic pain is common, complicating diabetes, shingles, HIV, and cancer. Medications are often ineffective or cause various adverse effects, so better approaches are needed. Half a century ago, electrical stimulation of specific brain regions (neuromodulation) was demonstrated to relieve refractory NP without distant effects, but the need for surgical electrode implantation limited use of deep brain stimulation. Next, electrodes applied to the dura outside the brain’s surface to stimulate the motor cortex were shown to relieve NP less invasively. Now, electromagnetic induction permits cortical neurons to be stimulated entirely non-invasively using transcranial magnetic stimulation (TMS). Repeated sessions of many TMS pulses (rTMS) can trigger neuronal plasticity to produce long-lasting therapeutic benefit. Repeated TMS already has US and European regulatory approval for treating refractory depression, and multiple small studies report efficacy for neuropathic pain. Recent improvements include “frameless stereotactic” neuronavigation systems, in which patients’ head MRIs allow TMS to be applied to precise underlying cortical targets, minimizing variability between sessions and patients, which may enhance efficacy. Transcranial magnetic stimulation appears poised for the larger trials necessary for regulatory approval of a NP indication. Since few clinicians are familiar with TMS, we review its theoretical basis and historical development, summarize the neuropathic pain trial results, and identify issues to resolve before large-scale clinical trials.

## CHRONIC NEUROPATHIC PAIN (NP) IS A MAJOR UNSOLVED HEALTH PROBLEM

Acute pain is evolutionarily advantageous—indeed it is critical for survival—because it warns us of harm and prompts us to flee from dangerous stimuli that threaten our survival. But if pain becomes chronic and dissociated from actual threats, the pain itself can threaten survival. Many patients with moderate or severe chronic pain have difficulty working, lose their jobs, and some become depressed, debilitated, and even impoverished. Relief of chronic pain is a major health priority since it is a problem of global scope that complicates virtually every type of disease and affects every organ system. A 2011 US Institute of Medicine (IOM) study reported that chronic pain is a major public health problem that affects more people than heart disease, cancer, and diabetes combined. It costs $560–$635 billion annually in health care costs and lost productivity in the US.^1^ There are two major types of pain: nociceptive pain caused by tissue damage (e.g. current injury or illness) and neuropathic pain (NP). Neuropathic pain is caused by prior injuries or illnesses that leave long-term damage to pain neurons, causing pain-sensing or transmitting neurons in the peripheral and/or central nervous system to fire action potentials despite the absence of a painful stimulus. Neuropathic pain is considered especially difficult to treat, because common pain relievers such as non-steroidal anti-inflammatories and opioids are less effective than for nociceptive pain, and disease-modifying treatments to repair neural damage are not yet available.

## THE DEVELOPMENT OF BRAIN STIMULATION TO TREAT CHRONIC PAIN

The medical field mostly relies on chemicals to treat illness, but since neurons use electrical signaling, electrical currents can alter their activity—a phenomenon increasingly exploited to treat neurological disorders. The first evidence for the use of electrical stimulation to treat chronic pain comes from antiquity, in the *Compositiones Medicam entorum*, the early guide to drugs and recipes written in 47 CE by Scribonius Largus, the court physician of the Roman emperor Claudius.^2^ He described using electrical currents to treat headaches and gout by applying electric torpedo fish to the painful regions. This treatment was popular for seizures, depression, and pain until the eighteenth century.

Electricity-based therapies later multiplied, based on the work of Luigi Galvani, Charles Le Roy, Duchenne de Boulogne, Beard and Rockwell, and others.^3^ Obviously, not all such treatments were well-grounded. Electrical stimulation was also applied to treat refractory chronic pain, with deep brain stimulation (DBS) as the first modern method. In DBS, small electrodes are surgically implanted in precise brain locations to deliver tiny electrical currents to neurons immediately adjacent to the electrode. Thus, unlike with medications, there are no distant adverse effects (e.g. rashes, gastrointestinal upset, allergies). Since only nearby neurons are affected, most brain functions continue unperturbed. A battery is implanted subcutaneously to power the electrode using technology based on cardiac pacemakers. A 1960 article by Heath and Mickle reported that DBS applied to the septum between the lateral ventricles of the brain produced immediate pain relief in a series of six patients with intractable pain, results duplicated by other early studies.^4^–^6^ In 1977, Richardson and Akil reported analgesic efficacy of DBS of the periaqueductal and periventricular gray matter.^7^,^8^ Stimulation of another deep target involved in pain sensation, the periventricular gray matter of the posterior thalamus, brought good pain relief to patients with cancer pain.^9^ Despite these encouraging results, high costs and rates of complication have limited DBS use; 3.9% of patients developed permanent neurological deficits, thalamic hemorrhage, or death, while 19.1% of patients had temporary complications, including neurological deficits, infection, and hardware malfunction.^10^

Epidural brain stimulation then emerged as a less invasive alternative. Here the electrodes are implanted under the skull, but outside the dura, so the brain itself is not disturbed and the risk is lower, although only superficial areas of the brain can be reached. Tsubokawa and colleagues first reported its efficacy in seven patients with thalamic pain syndromes.^11^ This group also compared the effects of stimulating various cortical regions on inhibiting the burst of hyperactivity of thalamic neurons that they associated with neuropathic pain.^11^ Better long-term inhibition of thalamic firing was induced by stimulating the motor cortex—more specifically, above the motor cortex site that corresponds to the painful area. Tsubokawa et al. then implanted electrodes over the motor cortex and longitudinally monitored 11 post-stroke patients with thalamic pain.^12^ A total of 73% (8/11) reported excellent pain control, which persisted unchanged in five patients (45%) for more than 2 years. Since then, various types of NP have been successfully treated with dural motor cortex stimulation (MCS), including post-stroke pain, spinal cord injury pain, thalamic pain, trigeminal neuralgia, trigeminal neuropathic pain, and trigeminal deafferentation pain (anesthesia dolorosa) syndromes.^13^ A recent meta-analysis of the various MCS trials found that 64% of patients with NP reported significant pain relief.^14^ The fact that up to 70% of these patients would undergo epidural MCS again provides additional evidence of clinical value.^15^

## PRINCIPLES OF NON-INVASIVE TRANSCRANIAL MAGNETIC STIMULATION (TMS)

The success of dural MCS inspired consideration of even less invasive stimulation modalities, and the best developed currently is transcranial magnetic stimulation (TMS). In TMS, a trained administrator holds an array of electrical coils at a precise location on the patient’s scalp overlying the target cortex. Capacitors are rapidly charged and discharged to pass brief electrical currents through the coils that in turn generate brief strong magnetic fields. These fields penetrate through nearby tissues, including the scalp, skull, meninges, and cerebrospinal fluid, to induce electric currents in underlying cortical neurons. The frequency of TMS pulses influences the effects on axons. Low frequencies of less than 5 Hz will hyperpolarize axons, transiently reducing their normal firing to inhibit their normal effects. This technique can be used to map brain functions for experimental reasons or, clinically, to help neurosurgeons identify eloquent areas of cortex to preserve during surgery. It is safer than the Wada test previously used for this purpose, and less dependent on patient cooperation than functional MRI.

In contrast, frequencies higher than 5 Hz—and typically 10 Hz is used—serve to depolarize the axolemma, and, if the current is sufficiently strong, this will trigger action potentials in nearby neurons. These then propagate along the axons towards their usual postsynaptic targets. The TMS magnetic fields only reach 2–3 cm into the cortex, and the spatial configuration of the affected area depends on the device properties, coil configuration, and axonal orientation. The most commonly used “figure-of-eight” coils can trigger action potentials in a ∼2-cm cone-shaped field.

Transcranial magnetic stimulation is applied differently for various experimental, diagnostic, and therapeutic uses. The use of low-frequency TMS to transiently inhibit cortical firing was mentioned above. Applying multiple excitatory TMS pulses to both motor cortices in the brain, and then subtracting the time for peripheral motor conduction by stimulating over the spinal cord, has long been used as a method for measuring the integrity of the central motor conduction pathways. Although this has largely been supplanted by MRI, at least in some cases, TMS measurements may be more sensitive.^16^

Single TMS stimulation of the motor cortex, in addition to inducing a muscle twitch in the corresponding muscle, also produces a subsequent period of electromyographic (EMG) suppression lasting up to 300 ms. This is termed the “cortical silent period” (CSP), and it is believed to reflect the transient refractoriness that follows every action potential. Exploration of CPS in neurological illnesses is contributing to our understanding of disease mechanisms.

“Paired pulse” is another TMS method used to assess experimentally how connections to the motor cortex influence its excitability. The first or “conditioning” stimulus is applied to a brain region of interest prior to a second “test” stimulus applied to the motor cortex. The effects of the first pulse on the motor response to the text stimulus provides an index of whether the region of interest has inhibitory, excitatory, or mixed modulatory connections with the motor cortex.

In contrast, repeated sessions of repetitive TMS (rTMS) are mainly used for therapeutic applications. In the EU and the US, rTMS of the dorsolateral prefrontal cortex using the Neuronetics NeuroStar TMS device (Neuronetics^®^ Inc., Malvem, PA, USA) and Brainsway’s Deep TMS device (Brainsway, Inc., Jerusalem, Israel) have US Food and Drug Administration (FDA) approval to treat refractory depression. There is lesser evidence of efficacy of rTMS in various other neurological conditions including bipolar disorder, schizophrenia, anxiety disorders, movement disorders, and rehabilitation from stroke.^17^ Different areas of the cortex are targeted in these different applications. Our focus here is to review the methods and evidence pertaining to treatment of chronic pain, which usually involves applying rTMS to the primary motor cortex (M1).

## METHODS OF APPLYING REPETITIVE TRANSCRANIAL MAGNETIC STIMULATION (rTMS) TO THE MOTOR CORTEX

Transcranial magnetic stimulation of the primary motor cortex is conducted with the patient in a reclining chair with support for the head and neck, while the operator stands behind and holds the TMS coil against the side of the patient’s head over the ear. The first task is to locate M1, which is done by monitoring the twitch evoked by TMS pulses. In its simplest form, this can be monitored visually, but most clinical applications involve adjusting the intensity of the administered TMS pulses so as not to evoke a motor response, which can be uncomfortable and impractical if hundreds of pulses are to be administered. Thus, electromyography equipment is usually interfaced with the TMS equipment and used to set the TMS pulse intensity to a sub-threshold value that is a fixed percentage (e.g. 80%–90%) of the patient’s resting motor threshold (RMT). Typically this procedure is repeated before each TMS session. The RMT is defined as the minimum stimulation intensity that elicits a motor response to 5 of 10 TMS pulses. The RMT depends on various factors, including the integrity of motor pathways and the tonic level of excitability in the muscle, as well as individual scalp-to-cortex distance and effect of pharmacological treatment.^17^

After assessing the RMT and setting the intensity, rTMS is applied in bursts of stimuli (“trains”) using a specific frequency and inter-train interval. The number of pulses delivered is usually between 500 and 2,500, and frequencies between 5 Hz and 20 Hz are used. Coil design and orientation are also important. Early coils were simple circles. The later “figure-of-eight” coil uses two circular coils to induce a stronger and more focal magnetic field at their intersection. Other newer designs include the tilted double-coil and the H-coil, which uses multiple loops to penetrate up to 8 cm or increase focality. Since the orientation of the magnetic field determines which neurons are affected, specific coil orientations are preferred when stimulating different brain regions.

## REVIEW OF THE STUDIES OF rTMS OF THE MOTOR CORTEX FOR CHRONIC PAIN

Studies involving one single application of rTMS to the motor cortex have provided proof of concept for efficacy against pain. Some involve experimental induction of brief pains in healthy volunteers (reviewed in Mylius et al.^18^) or in patients with chronic pain. These studies were used to compare efficacy of different stimulation sites, specifically the primary and secondary motor cortices, dorsolateral prefrontal cortex, the primary and secondary somatosensory cortices, and the supplementary and premotor areas.^18^ As with epidural stimulation, stimulating the primary motor cortex generally provided the best pain relief. In contrast, depression is best treated by applying rTMS to the dorsolateral prefrontal cortex—additional evidence of different anatomical substrates for NP and depression. The fact that motor but not sensory cortex stimulation relieves pain is not yet understood. Although TMS only directly affects the superficial cortex since the currents rapidly dissipate,^19^ the action potentials triggered propagate to influence distributed neural networks. Effects of motor cortex stimulation on chronic pain are thought to involve M1 projections to pain-modulating structures; perhaps among them are the medial thalamus, anterior cingulate/orbitofrontal cortices, and the periaqueductal gray matter (PAG).^17^ The incertothalamic pathway has recently been implicated in rats, with demonstration that TMS of M1 increases electrical activity in the zona incerta, which projects to and inhibits activation of the posterior thalamus.^20^

The more clinically relevant studies involve administering rTMS to patients with clinical chronic pain conditions. We identified 24 publications between 2001 and 2013 that assessed efficacy of rTMS for treating chronic pain. Among them, 15 assessed the effects of a single session only of TMS (Table 1). While 12/15 reported pain relief, the effects of a single rTMS session are transitory and therefore inadequate for clinical management of chronic pain, so their relevance for clinical practice is limited. Table 2 summarizes the nine studies that evaluated the effects of multiple rTMS sessions on chronic pain. Four studies used five consecutive days of treatment, and five involved two consecutive weeks of five sessions of weekday TMS. Among them, 6/9 showed significant pain reduction. Importantly, it was found that consecutive sessions of weekday rTMS extended the effects of a single session of rTMS to produce residual pain relief that can persist even after rTMS is discontinued, which is the cornerstone of clinical benefit.^17^ Publications report that these residual effects can last up to two weeks, but in clinical use, some patients are able to maintain pain relief with once-monthly sessions of rTMS, so this requires better characterization. The mechanisms are not known but presumably involve neuronal plasticity, such as that triggered by other situations involving repeated neuronal firing. Accordingly it is suggested that maintenance therapy, which consists of a priming week or weeks, of daily weekday rTMS sessions, followed by maintenance sessions at longer intervals, will maintain long-lasting effects. To date, only one study of 40 fibromyalgia patients assessed long-term rTMS maintenance therapy.^41^ The protocol comprised one priming week of daily weekday rTMS, then one session weekly for 3 weeks, three sessions at fortnightly intervals, followed by three monthly sessions; TMS ended at week 21. Reduced pain intensity and improved quality of life measures were demonstrated between day 5 through week 25, 4 weeks after the TMS stopped.^41^

**Table 1:** Studies Assessing Effects of One Session of Repetitive Transcranial Magnetic Stimulation (rTMS) of the Motor Cortex on Chronic Pain.

**Population Studied**	**Number Studied**	**Significant Effects**	**Frequency of TMS (Hz)**	**Intensity of TMS (% RMT)**	**Number of TMS Pulses**	**Citation Number**
Mixed NP	14	+	10	80	1000	21
Mixed NP	18	+	10	80	1000	22
Mixed CP	12	−	20	80	800	23
Mixed NP	60	+	10	80	1000	24
CRPS	10	+	10	110	120	25
Mixed NP	12	−	20	90	1600	26
Mixed NP	20	+	5	90	500	27
Mixed NP	27	−	5	95	500	28
Mixed NP	22	+	10	90	1200	29
Mixed NP	13	+	10	90	500	30
Mixed NP	28	+	20	90	1600	31
Mixed NP	46	+	10	90	1200	32
Post-stroke pain	20	+	5	100	500	33
Mixed NP	14	+	10	90	2000	34
SCI	16	+	10	110	2000	35

Population: Mixed NP, mixed neuropathic pain patients; Mixed CP, mixed chronic pain patients; CRPS, complex regional pain syndrome; SCI, spinal cord injury; TMS, transcranial magnetic stimulation. Significant effects: + represents significant reduction in pain score following transcranial magnetic stimulation (TMS) treatment. Numbers presented in the frequency, intensity, and number of pulses represent the higher values in case of more than one condition.

**Table 2:** Studies Assessing Effects of Multiple Sessions of Repetitive Transcranial Magnetic Stimulation (rTMS) of the Motor Cortex on Chronic Pain.

**Patients**	**Population**	**Significant Effects**	**Frequency of TMS (Hz)**	**Intensity of TMS (% RMT)**	**Number of TMS Pulses**	**Number of Treatment Days**	**Citation Number**
Mixed NP	48	+	20	80	2000	5	36
SCI	12	−	5	115	500	10	37
FM	30	+	10	80	2000	10	38
SCI	13	−	10	80	1000	5	39
CRPS	23	+	10	100	2500	10	40
FM	40	+	10	80	1500	5^*^	41
FM	15	+	10	80	2000	10	42
DPN	25	+	20	100	1500	5	43
Mixed NP	70	+	5	90	500	10	44

* In Mhalla et al. 2011^41^ five treatment days were followed by a maintenance regime.

Population: Mixed NP, mixed neuropathic pain patients; FM, fibromyalgia; CRPS, complex regional pain syndrome; SCI, spinal cord injury; DPN, diabetic polyneuropathy. Significant effects: + represents significant reduction in pain score following transcranial magnetic stimulation (TMS) treatment. Numbers presented in the frequency, intensity, and number of pulses represent the higher values in case of more than one condition.

Since the TMS treatment parameters varied among the published studies it is difficult to determine which specific parameters are best for clinical use. Complicating matters further, only 10 of 24 studies recruited homogeneous populations of patients, precluding certainty about which conditions are most responsive to TMS. Homogeneous studies have been published on complex regional pain syndrome (CRPS),^25^,^40^ spinal cord injury (SCI),^35^,^37^,^39^ diabetic polyneuropathy (DPN),^43^ post-stroke pain,^33^ and fibromyalgia.^38^,^41^,^42^ Most studies had small sample sizes, with only four recruiting more than 40 patients (mean = 25, median = 20). Another limitation is the variation in applied stimulation parameters such as frequency (ranged from 5 Hz to 20 Hz), intensity of RMT (ranged from 80% to 115%), and total number of pulses (ranged between 120 and 2,500). A 2010 Cochrane Systematic Review concluded that higher stimulation frequencies (>5 Hz), greater numbers of stimuli (>500), and multiple sessions (>1) yielded better results.^45^ The contribution of many TMS factors, including coil orientation, duration of each pulse train, inter-train interval, and number of trains, is not yet understood.

An additional unresolved question concerns which site within the motor cortex yields the strongest benefit for pain patients. Most studies stimulated the motor cortical representation of patients’ painful site, but one suggested that stimulating adjacent motor cortex sites yields better analgesia.^24^ Placebo effects also need to be better addressed. These are considerable in both pain trials and device trials. However, given that TMS evokes both visual, auditory, and tactile sensations, sham procedures are difficult to design, and there is no consensus regarding the best design of a true double-blinded, sham-controlled study, since researchers and often subjects can usually distinguish between real and sham devices.^45^ Some methods of sham TMS offer visual verisimilitude, e.g. inert or inactivated TMS coils, but fail to produce auditory and electrical sensations.

## SAFETY CONCERNS PERTAINING TO MOTORCORTEX rTMS TREATMENT OF NP

Although the big advantages of TMS are its non-invasiveness and lack of extracranial effects, there are safety considerations, particularly when many TMS pulses are applied repeatedly, as required for clinical effects. Detailed safety guidelines established at a 2009 global consensus conference of experts establish absolute and relative contraindications to TMS.^46^ Like MRI, TMS is absolutely contraindicated for people with ferromagnetic implants in or near the head, including shrapnel or medical implants, because magnetic fields might cause the metal to move or overheat. Magnetic pulses can also cause electronically controlled devices to malfunction or fail.

In patients without intracranial ferromagnetic implants, the only potentially serious complication of TMS is the possibility of inducing a single seizure. This is an expected consequence of triggering action potentials in cortical neurons. Therefore, TMS is relatively contraindicated and, in most cases, should not be administered to patients with increased seizure risk, for instance those with epilepsy or epileptogenic brain lesions (e.g. strokes or tumors), or taking medications that increase seizure risk (some antibiotics, antivirals, antidepressants and other psychiatric medications, illicit drugs, and alcohol).For others, the risk of a TMS-induced seizure is very low, estimated at ≤1/10,000.^46^ As with other types of environmentally provoked seizures, e.g. those triggered by hypoglycemia, there is no evidence that a single provoked seizure can or will trigger epilepsy. The only common adverse effect of TMS is headache, which is reported by about 1 in 10 subjects. This is attributed to the TMS coil being pressed against subjects’ heads for an extended time. The TMS-induced headaches are usually mild and respond to usual headache treatments such as acetaminophen. Lastly, depending on where TMS is applied, and its intensity, suprathreshold application to the motor cortex can activate the facial, trigeminal, or auditory nerves to cause discomfort. As with MRI, people undergoing TMS are usually offered earplugs to minimize exposure to the noises generated by the TMS coils.

## RECENT TECHNOLOGICAL ADVANCES AND RECOMMENDATION FOR FUTURE RESEARCH

Over the years TMS pulse generators have not changed significantly, but new coil designs and cooling units allow more TMS pulses to be administered at higher frequencies and more focally. Cooled coils prevent coils from overheating after a long series of pulses. Innovative coil configurations provide greater focality and deeper penetration. Brainsway’s H-coil allows deeper penetration of TMS pulses. It obtained EU approval to treat major depressive disorder in 2008, bipolar depression in 2009, schizophrenia in 2010, and post-traumatic stress disorder in 2011. In January 2013, Brainsway won US and Canadian approval to market its Deep TMS device for drug-resistant depression.^47^ Home-based rTMS systems are currently in development.^48^

**Figure 1:**
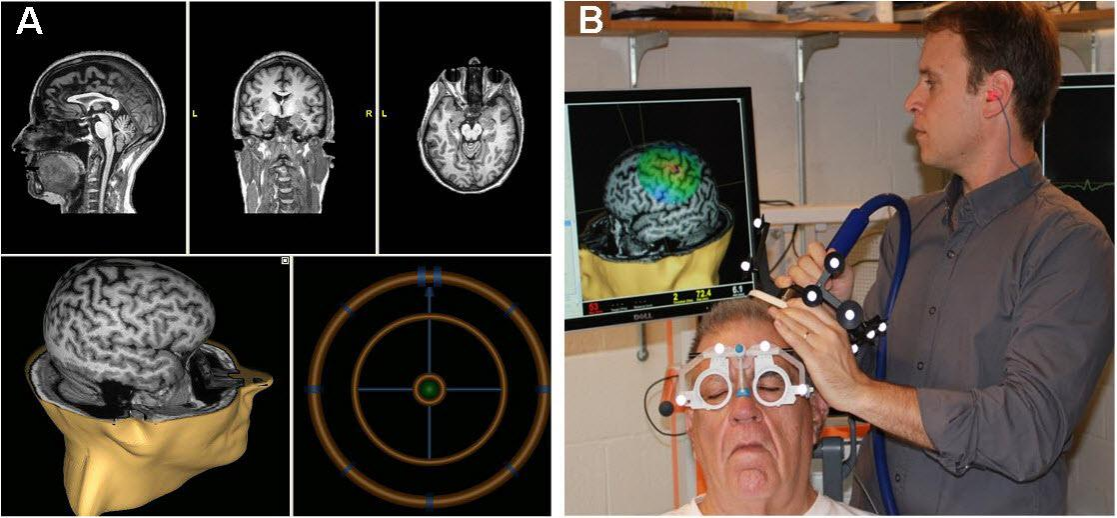
**The MRI-navigated Nexstim Interface.** A: Screen shot of the Nexstim neuronavigation interface: The top three panels represent the sagittal, coronal, and axial (left to right) MRI views used to locate specific spatial landmarks relative to the stereotactic spheres (shown in panel B) that are used for neuronavigated TMS. The lower panels display the stimulation target on a 3D MRI (left) and the bulls-eye target (right) that ensures that the operator holds the coil to the patient’s scalp at the correct location and orientation. B: The operator holds the figure-of-eight coil to the patient’s scalp while monitoring the brain stimulation site on the 3D MRI. Note the stereotactic spheres mounted on the coil that identify its position relative to the spheres on the patient’s goggles that localize the patient’s head.

**Figure 2:**
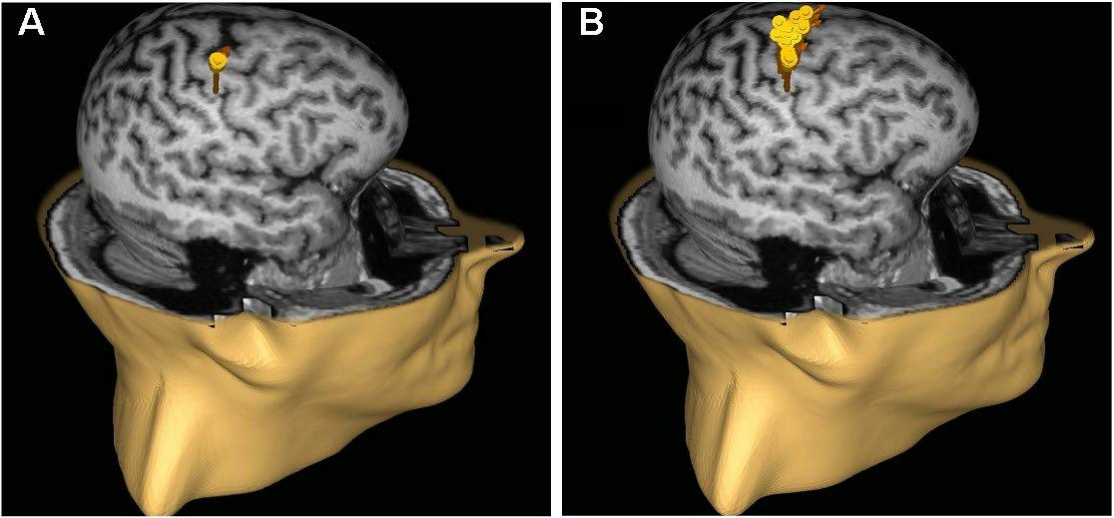
**MRI-guided Neuronavigation Allows rTMS to Target the Same Cortex More Precisely and Reproducibly.** Panels A and B display a repetitive application of 20 stimuli with (A) and without (B) neuronavigation to the motor cortex area. Note the accuracy of the neuronavigated system (A), compared to the lessprecise application achieved without MRI navigation.

Another significant development, introduced more than a decade ago, is “neuronavigated” TMS. Several companies developed “frameless stereotactic” systems (Figure 1) that use infrared cameras to register position and orientation of the TMS coil relative to the subject’s head, and integrate individual cortical topography from each person’s head MRI, to guide placement of the TMS coil. This informs the TMS administrator about the actual location of the desired brain target in that person and also enables placing the coil at precisely the same spot during different TMS sessions. Computer modeling shows that MRI-navigated TMS reduces variability in induced current compared to hand-held TMS, and more precisely and reproducibly locates motor cortex targets in human patients.^49^–^51^ Nexstim’s Navigated Brain Stimulation system won FDA approval for presurgical mapping of cortical function in 2010. Importantly, almost all studies of rTMS for pain used hand-held coils, meaning that the location of stimulation likely varied slightly during successive sessions, as illustrated in Figure 2. Additional study is required to determine if MRI-navigated TMS improves outcomes.

In summary, TMS is non-invasive and requires no anesthesia or sedation, has no known long-term adverse or systemic side effects in properly selected patients, and offers an alternative to medications for relieving neuropathic pain. Although the small studies reviewed here often demonstrate benefit, their methodological limitations make them insufficient for obtaining regulatory approval. These include too few subjects with diverse causes of pain, inconsistency in stimulation targets and TMS parameters, and insufficient sham and blinding. Future adequately powered studies in homogeneous populations should help clarify whether MRI-navigated TMS or advanced coil designs offer added benefit for neuropathic pain, which cortical locations to target, and best stimulation parameters to use for randomized clinical trials. This might allow a promising experimental pain treatment to transition from the research laboratory into clinical practice.

## Abbreviations:

CP: chronic pain;
CRPS: complex regional pain syndrome;
DBS: deep brain stimulation;
DPN: diabetic polyneuropathy;
EBS: epidural brain stimulation;
EMG: electromyography;
FDA: Food and Drug Administration;
HIV: human immunodeficiency virus;
IOM: Institute of Medicine;
MCS: motor cortex stimulation;
MRI: magnetic resonance imaging;
NP: neuropathic pain;
PAG: periaqueductal gray matter;
RMT: resting motor threshold;
rTMS: repetitive transcranial magnetic stimulation;
SCI: spinal cord injury;
TMS: transcranial magnetic stimulation;
US: United States.
